# Characterizing the psychiatric profile of the patients with phenylketonuria

**DOI:** 10.1192/j.eurpsy.2026.12018

**Published:** 2026-07-31

**Authors:** H. C. Ozden, T. Oz, A. B. Kahraman, E. Ozcelik Eroglu, Y. Yildiz, A. Tokatli, Y. Ayhan

**Affiliations:** 1 Department Of Psychiatry, Hacettepe University Medical School; 2 Department Of Psychiatry, Etlik City Hospital, Ankara; 3 Department of Pediatrics, Division of Pediatric Metabolism, Konya City Hospital, Konya; 4 Department of Pediatrics, Division of Pediatric Metabolism, Hacettepe University Medical School, Ankara, Türkiye

## Abstract

**Introduction:**

Inborn errors of metabolism (IEM) are mostly autosomal-recessive and prevalent in Türkiye due to consanguinity. IEMs can affect the CNS and present as psychiatric disorders; low awareness may obscure psychiatric and cognitive problems.

**Objectives:**

Hacettepe University is a national referral center where IEM patients are followed lifelong every 3–6 months. From this large cohort, we aimed to characterize psychiatric profiles.

**Methods:**

All ≥18-year patients with IEMs attending Hacettepe University Department of Metabolism who consented (Jul 2021–Jun 2022) were included. Participants were asked to complete the Beck Depression Inventory (BDI) and the Beck Anxiety Inventory (BAI); the Adult ADHD Scale (Turgay) and the Reading the Mind in the Eyes Test (RMET) were administered. Of 90 referred, 47(52.2%) had PKU; the remaining 43 had other 19 IEMs, therefore analyses focused on PKU first. We will characterize the psychiatric profiles of the other IEMs as the sample size increased over time. All statistical analyses were conducted in Jamovi (v2.3.28.0); significance was set at *p*<0.05.

**Results:**

Of the participants diagnosed with PKU, 27(57.4%) were women and 20(42.6%) were men. The mean age was 24.7±5.3 years. Education levels were: primary school 1(2.4%), middle school 6(14.6%), high school 23(56.1%), and university 11(26.8%). Of the participants, 38(80.9%) were diagnosed via newborn screening and 9 (19.1%) during postnatal examinations. All patients attended regular follow-ups and their disease was well controlled. Two patients (4.3%) reported alcohol use, three (6.5%) were current smokers, and 22(47.8%) had previously sought psychiatric care for mental health complaints.

On the BDI, 42.1% of respondents scored above the cutoff for mild depression. When participants were grouped by being above or below the cutoff, the number of women above the cutoff was higher than the number of men (Table 1).

On the BAI, 7(20.0%) scored in the mild anxiety range, 6(17.1%) in the moderate range, and 2(5.7%) in the severe range. Overall, 22.8% scored ≥16, the value considered positive for screening. There was no sex difference in BAI scores (p=0.987).

None of the participants with PKU met symptom criteria for ADHD. Mean RMET score was 20.8±4.25. Relative to the Turkish validation norm of 23.64±3.38, this corresponded to z = −0.84. The difference was significant (t(37) = −4.12, p<0.001).

**Table 1.** Distribution of BDI Categories by Sex Table 1. Long description.

	Depressed		
Sex	Y	N	Total
Women	13 (56.5%)	10 (43.5%)	23 (100.0%)
Men	3 (20.0%)	12 (80.0%)	15 (100.0%)
Total	16 (42.1%)	22 (57.9%)	38 (100.0%)

Chi-Square Tests (N=38)			
	Value	df	P
χ²	4.97	1	0.026

**Conclusions:**

In PKU, depression risk is high (higher in women), about one quarter show elevated anxiety. Despite the absence of ADHD and education levels comparable to the general population, the observed impairment in theory-of-mind abilities is notewotrthy. Psychiatric profiling in IEMs is necessary.

**Disclosure of Interest:**

None Declared

## Long descriptions

### Table 1. Long description

The first section is a contingency table for Depressed status (Y or N) by Sex.

- Women: 13 (56.5 percent) are Y, 10 (43.5 percent) are N, totaling 23 (100.0 percent).

- Men: 3 (20.0 percent) are Y, 12 (80.0 percent) are N, totaling 15 (100.0 percent).

- Total: 16 (42.1 percent) are Y, 22 (57.9 percent) are N, totaling 38 (100.0 percent).

The second section provides Chi-Square Tests for N equals 38:

- The Chi-Square symbol value is 4.97.

- The d f (degrees of freedom) is 1.

- The P value is 0.026.

Navigate back to Table 1..

